# Computational analysis of RNA methyltransferase Rv3366 as a potential drug target for combating drug-resistant *Mycobacterium tuberculosis*

**DOI:** 10.3389/fmolb.2023.1348337

**Published:** 2024-01-11

**Authors:** Tasmin Nazim, Vipul Kumar, Faraz Ahmed, Nasreen Z. Ehtesham, Seyed E. Hasnain, Durai Sundar, Sonam Grover

**Affiliations:** ^1^ Department of Molecular Medicine, Jamia Hamdard, New Delhi, India; ^2^ Department of Biochemical Engineering and Biotechnology, Indian Institute of Technology Delhi, New Delhi, India; ^3^ Department of Life Sciences, School of Basic Science and Research, Sharda University, Greater Noida, India

**Keywords:** Droxidopa, Levodopa, MDR, MTases, TB, XDR

## Abstract

*Mycobacterium tuberculosis* (*M.tb*) remains a formidable global health threat. The increasing drug resistance among *M.tb* clinical isolates is exacerbating the current tuberculosis (TB) burden. In this study we focused on identifying novel repurposed drugs that could be further investigated as potential anti-TB drugs. We utilized *M.tb* RNA methyltransferase Rv3366 (spoU) as a potential drug target due to its imperative activity in RNA modification and no structural homology with human proteins. Using computational modeling approaches the structure of Rv3366 was determined followed by high throughput virtual screening of Food and Drug Administration (FDA) approved drugs to screen potential binders of Rv3366. Molecular dynamics (MD) simulations were performed to assess the drug-protein binding interactions, complex stability and rigidity. Through this multi-step structure-based drug repurposing workflow two promising inhibitors of Rv3366 were identified, namely, Levodopa and Droxidopa. This study highlights the significance of targeting *M.tb* RNA methyltransferases to combat drug-resistant *M.tb*. and proposes Levodopa and Droxidopa as promising inhibitors of Rv3366 for future pre-clinical investigations.

## Introduction

*M.tb* is the causative agent of TB and still one of the world’s lethal infectious pathogens. According to the latest 2023 WHO Global Tuberculosis Report, during 2022, an estimated 10.6 million individuals globally contracted tuberculosis (TB), with a range of uncertainty between 9.9 and 11.4 million cases. This figure translates to about 133 new cases per 100,000 individuals in the population. Among these newly reported TB cases, 6.3% were identified in individuals living with HIV and 1.13 million people lost their lives due to this co-infection. The majority of TB cases in 2022 were concentrated in specific WHO regions, with South-East Asia comprising 46%, followed by Africa with 23%, and the Western Pacific with 18%. Smaller portions were observed in the Eastern Mediterranean (8.1%), the Americas (3.1%), and Europe (2.2%) (WHO, 2023). An increase in the TB incidence rate was observed partly due to the continuously emerging new drug resistant *M.tb* strains and partly because of the COVID-19 pandemic (Shariq et al., 2022; Bagcchi, 2023). Unfortunately, the COVID-19 pandemic has imposed a detrimental impact on the progress made in the fight against TB before 2019. It has worsened the overall burden of the disease by disrupting routine TB diagnosis and treatment (Chakaya et al., 2020; Chakaya et al., 2021; Sheikh et al., 2022). Understanding the reasons of drug resistance in *M.tb* clinical isolates would eventually lead to the identification of new drug targets as well as development of effective anti-TB drugs. The current standard TB regimen, involving drugs like Rifampicin and Isoniazid, faces poor compliance, contributing to the problem of resistance. While new drugs like Bedaquiline and Delamanid show promise against MDR and XDR-TB, resistance issues have been observed in clinical practice (Sullivan and Ben Amor, 2016). Consequently, there is a critical need for research into new anti-TB drugs. To combat the exacerbated virulence and resistance pattern of the pathogen, and to eliminate TB globally by 2035, Researchers have explored repurposing existing drugs to address this challenge, focusing on finding new uses for approved drugs to quickly transition them from lab to patient care (Sheikh et al., 2022; Malik and Sinha, 2023; Singh et al., 2023). Currently there are 5-6 repurposed drugs approved by WHO for the treatment for drug resistant TB, namely: delamanid, bedaquiline, pretomanid, clofazimine, carbapenems, and linezolid (Silva et al., 2018; Sharma et al., 2023; Singh et al., 2023).

In all living organisms RNAs, tRNAs, mRNAs, long noncoding RNAs and microRNAs undergo almost 160 different chemical modifications (Boccaletto et al., 2018; Xuan et al., 2018). In case of microbes, methylation of RNA is a crucial modification for the regulation of its stability, processing, nucleus-cytoplasmic export and translation (Meyer and Jaffrey, 2014; Wang et al., 2014; Slobodin et al., 2017; Anderson et al., 2018; Hu et al., 2021). The process of methylation is carried out by a distinct family of proteins called methyltransferases (MTases). Methyltransferases are like multitasking conductors in the cell orchestra, involved in crucial activities such as creating molecules, relaying signals, fixing proteins, managing DNA packaging, and even silencing genes (Petrossian and Clarke, 2011). They’re essential players in controlling how genes express themselves and shape the fate of the cell. For instance, Rv3366, a SAM-dependent Methyltransferase in *M.tb*, plays the role of transferring methyl groups from SAM (S-adenosylmethionine) to RNA, specifically tweaking its structure. At present, Methyltransferase activity of Rv3366 on *M.tb* RNA has been studied *in vitro* recently which will be communicated in addition to its further characterization. Limited mechanism of action has been revealed about this gene of *M.tb*.

When we look closely at the types of RNA modifications, we see that a large chunk—around 56%—is handled by RNA methyltransferases, followed by tRNA methyltransferases at about 39%. These modifications are vital for the growth and survival of the cell. In the game of antibiotics versus bacteria, the primary targets are not the proteins but the RNAs (Malke et al., 1990). Antibiotics like aminoglycosides, tetracycline, and others take aim at the RNA-rich surfaces of ribosomal subunits, disrupting protein production. Any changes to these rRNAs, like methylations or alterations in their building blocks, could lead to resistance against these drugs (Poehlsgaard and Douthwaite, 2005).

This makes RNA methyltransferases potential new targets in fighting infectious diseases like tuberculosis. They could specifically modify the rRNAs of the infectious agent, inhibiting protein synthesis and also thwarting the mechanisms that make the bacteria resistant to drugs. Scientists have been exploring drugs that target these methyltransferases, aiming to find ones that can specifically tackle the rRNA modifications in tuberculosis and other infectious diseases (Foik et al., 2018). In human, rRNA and tRNA MTases collectively cover about 95% of total MTases where rRNA MTases constitute a significant portion with approximately 56% having unique enzymatic functions (Lennard, 2010). This indicates their essentiality in cell growth and survival. One of the important options to investigate as possible therapeutic targets in *M. tuberculosis* is methyltransferases (MTases). It is a large, diverse, and biologically relevant protein superfamily that uses S-adenosyl-L-methionine (SAM) to methylate various biomolecule substrates, including proteins, DNA, and RNA. Previous research has demonstrated that *M.tb* contains a very high number of 121 distinct MTases (Ali et al., 2021; Rani et al., 2022). In comparison, MTases make up about 1.2% of all gene products in yeast (Petrossian and Clarke, 2009; Petrossian and Clarke, 2019). Also, there are 17 functional methyltransferases in *Helicobacter pylori* (Lee et al., 2015). However, while many organisms have multiple methyltransferases, not all methyltransferases are active all the time. It was found that out of three putative methyltransferases in *Enterococcus faecalis* only one was active under the conditions tested (Huo et al., 2015).

Moreover, the differences in MTases protein structure between prokaryotes and eukaryotes make them ideal for investigating as drug targets. By inhibiting rRNA methyltransferases, it may be possible to address the antibiotic resistance in *M.tb* without interfering with host protein synthesis pathways (Salaikumaran et al., 2022). Among these RNA MTases, *M.tb* Rv3366 (SpoU) is a known tRNA/rRNA methylase which belongs to the RNA methyltransferase TrmL family (InterPro, 2022). The homologue of this protein is absent in human genome but present in various species like *Escherichia coli* (Gustafsson et al., 1996; Persson et al., 1997), *Aquifex aeolicus* (Hori et al., 2003), *Thermus thermophilus* (Hori et al., 2002), and as trm3 in *Saccharomyces cerevisiae* (Cavaillé et al., 1999). Among all these mentioned organisms SpoU has been reported to play a crucial role in tRNA modification. In case of *M.tb,* tRNA modification results in antibiotic resistance (Babosan et al., 2022).

The rise of resistance against existing drugs calls for a deeper search of a novel drug target as well as a novel drug to combat such extensive resistance by the *M.tb* pathogen. Considering the known fundamental importance of SpoU MTase in various organisms we selected *M.tb* Rv3366 as a potential drug target for computational analysis. The analysis of different MD simulation paths with different molecules showed that the flexibility in RNA methyltransferase contributes to its conformational stability, which plays a critical role in the development of a drug target against antibiotic resistance. This research sets the stage for creating effective anti-TB drugs by targeting this mechanism. Also, molecular docking and simulation studies conducted to explore the impact of Methyltransferase on drug-binding revealed a correlation between the docking scores and molecular simulation values for protein-drug complexes. The binding energy and increase in H-bonds confirms the stability of the drug target. By utilizing computational based drug repurposing approaches this study aims to investigate the high throughput interaction of Rv3366 with FDA approved drugs in order to screen for its potential inhibitors. This study can further help the researchers in finding novel repurposed drugs against drug resistant *M.tb*.

## Methods

### Computational methods

#### Protein structure retrieval, modeling and preparation

The RNA methyltransferase (Rv3366) (Uniprot ID: O50394) structure was modeled using Alphafold2 integrated in ColabFold with high confidence scores (pLDDT) for all the residues (>90), except for residues 38–41 and residue 56 it was between 70 and 90, for residues 42–55 between 50 and 70 (Jumper et al., 2021). The pLDDT score and Predicted aligned error matrix has been shown in Supplementary Figure S1 (Mirdita and Schütze, 2022). The model was then prepared using Schrodinger’s protein preparation wizard, which involved adding missing hydrogens, completing side chains and loops, and removing water molecules (Desmond 2020). The structure was refined with H-bond assignment at pH 7.0 using the PROPKA tool (Søndergaard et al., 2011), followed by restrained minimization using the OPLS3e forcefield (Roos et al., 2019) until heavy atoms converged to 0.3 Å. Subsequently, classical molecular dynamics (MD) simulations were conducted for 50 ns to equilibrate the structure.

For MD simulations, the system was solvated with TIP3P water in a periodic boundary box, ensuring a 10 Å distance between the protein complex and the box wall to prevent unwanted interactions. The system was neutralized by adding Na+/Cl− ions based on the complex’s charge, and a low-temperature (10 K) Brownian motion MD simulation was performed for 100 ps in the NVT ensemble to eliminate steric clashes and unfavorable conformations. The system was further equilibrated in both NVT and NPT ensembles using the Desmond Schrodinger suite’s “relax model system before simulation” option. Finally, unrestrained MD simulations were carried out for 50 ns in the NPT ensemble at 300 K and 1 atm, using the Nose–Hoover chain thermostat and Martyna–Tobias–Kelin barostat, with a time step of 2 fs and a recording interval of 50 ps. The resulting structure from the simulations was used for subsequent analysis (Desmond 2020).

#### Receptor grid generation and virtual screening workflow to screen ligands

**FIGURE 1:**
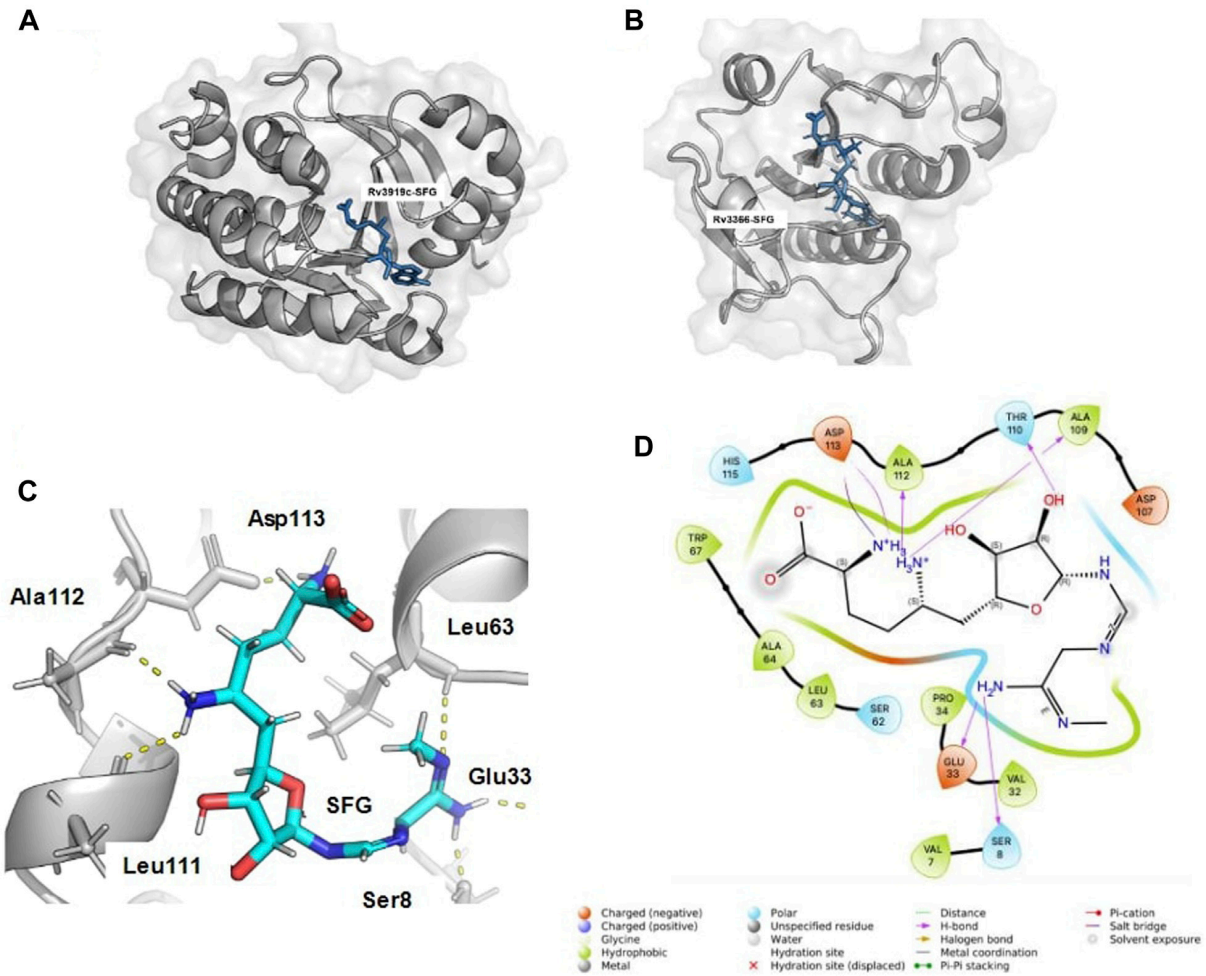
The binding alignment of SFG with Rv3366. **(A)** The native pose of SFG with Rv3919c (PDB ID: 7CFE). **(B)** The binding pose of SFG with Rv3366, when binding site alignment tool was used to find the binding site at Rv3366. **(C)** The docked pose of SFG with Rv3366 showing the hydrogen bond interactions **(D)** The 2D interaction diagram of Rv3366-SFG showing all kind of interactions.

In the receptor grid generation and virtual screening workflow, the binding site coordinates of the known inhibitor SFG were employed to create a grid (10 Å^3) for molecular docking. The SFG-bound structure of Rv3919c (PDB ID: 7CFE) was aligned with Rv3366’s binding site using Schrodinger’s Align Binding Site module. This module automatically detects the bound ligand and aligns the residues within 5 Angstrom from the ligand. Figure 1A shows the binding pose of SFG with Rv3919c (PDB ID: 7CFE), and Figure 1B shows the binding site alignment of SFG with modelled Rv3366. Once the grid was generated, ligands were retrieved from a set of FDA-approved compounds (2,499) obtained from DrugBank. These compounds were filtered based on Lipinski’s rule, resulting in 1,657 ligands. The LigPrep module of Schrodinger was then used to prepare the ligands, including neutralization, desalting, and minimization with the OPL3e force field (Friesner et al., 2006; Harder et al., 2016).

The virtual screening workflow comprised three steps: (i) High-Throughput Virtual Screening (HTVS), which docked the ligands flexibly and performed post-docking minimization, resulting in 500 FDA-approved drugs for the next step; (Migliori, Sotgiu et al.); Glide SP (Standard Precision), applied to the top 200 compounds from HTVS; and (iii) Glide XP, applied to the output compounds from Glide SP, with the top 50 FDA-approved compounds selected for further analysis (Friesner et al., 2006). The 3D visualization of docked complexes was done through ChimeraX (Pettersen et al., 2021).

#### Molecular mechanics/generalized born surface area (MM/GBSA) binding free energy

The results from the Virtual Screening Workflow, which included the docking of 50 FDA-approved drugs to their target proteins, were further analyzed to identify the top 20 molecules based on their MM/GBSA binding free energy computation through the following equations (Genheden and Ryde, 2015):

$$\text{MM}/\text{GBSA }\Delta \text{Gbind}=\Delta \text{Gcomplex }-\;\left(\Delta \text{Greceptor}+\Delta \text{Gligand}\right)$$

ΔG=ΔEgas+ΔGsol − TΔSgas

ΔEgas=ΔEint+ΔEelec+Δevdw

ΔGsol=ΔGgb+ΔGsurf

The gas-phase interaction energy (ΔEgas) was determined by summing the electrostatic (ΔEelec) and van der Waals (ΔEvdw) interaction energies, with the internal energy being disregarded. The solvation free energy (ΔGsol) comprised both non-polar (ΔGsurf) and polar solvation energy (ΔGgb), calculated using the VSGB solvation model and the OPL3e force field (Li et al., 2011; Edward et al., 2016). However, the entropy term was not considered in this calculation. In summary, the MM/GBSA binding free energy computation involved dissecting the energy contributions from various components, such as gas-phase interactions and solvation effects, to assess the binding stability of the molecules under investigation. In essence, MM/GBSA binding free energy is a commonly used method to establish a correlation between the binding energy of a compound and its experimental affinity for the target protein. It is important to note that the reported binding energy values are not absolute due to inherent limitations in accurately calculating force fields and entropy. However, it is generally accepted that a more negative value for the binding energy indicates a higher affinity of the compound for the target protein.

#### Molecular dynamics (MD) simulations of the top screened molecules

A total of eight FDA-approved drugs, as highlighted in the table, were selected based on their availability and functionality for further investigation with Rv3366. Molecular dynamics (MD) simulations of these selected complexes were conducted using the same protocol as outlined previously for the modeled protein. The primary objective of these simulations was to assess the stability of the top docked ligands and monitor their crucial interactions throughout the simulation. To comprehensively evaluate the stability and dynamic behavior of these complexes during the MD simulations, several structural metrics were calculated: (i) Root Mean Square Deviation (RMSD): RMSD measures the deviation of the ligand-protein complex from its initial structure over the course of the simulation. It provides insights into the overall stability and structural changes of the complex (Migliori et al., 2012). Root Mean Square Fluctuation (RMSF): RMSF quantifies the fluctuation of individual atoms or residues within the complex during the simulation. This analysis helps identify regions of the protein or ligand that exhibit high flexibility or interactions that may change significantly during the simulation. (iii) Radius of Gyration (Harder et al., 2016): Rg is a measure of the compactness ligand during the simulation. It offers information about the flexibility (Anderson et al., 2018). Solvent Accessible Surface Area (SASA): SASA calculation estimates the surface area of ligands that is accessible to solvent molecules throughout the simulation. Changes in SASA reveal alterations in the complex’s accessibility and potential conformational rearrangements. Furthermore, to gain insights into the critical interactions between the ligands and Rv3366, simulation interactions were examined. This analysis involved monitoring the types of interactions (such as hydrogen bonds, hydrophobic interactions, and electrostatic interactions) formed between the ligands and the protein throughout the simulation. The occupancy of these interactions was also calculated, indicating how frequently they occurred during the simulation (Schrödinger 2020).

## Results

### The top screened FDA-approved compounds against Rv3366

The DrugBank was used for structure-based virtual screening against Rv3366 using the virtual screening workflow of the Glide Schrodinger suite. The compounds were initially prepared and filtered in the workflow based on the Lipinski rule of five. Further, based on the virtual screening, the top 50 FDA-approved drugs were screened as top docked molecules (Supplementary Table S1). Then these docked complexes were used for further extensive screening using MM/GBSA binding free energy (Supplementary Table S2). Given the time and computational energy expense, simulation of all these 20 complexes was not feasible. Therefore, based on the already known functions of these screened FDA drugs, eight drugs, namely, Kappadione, Pamidronic acid, Pyridoxal Phosphate, Cedrazuridine, Levodopa, Droxidopa, Pyrophosphoric acid and Zoledronic acid, were selected against Rv3366. Based on this study through molecular docking and molecular dynamics simulations, out of eight drugs only two (Levodopa and Droxidopa) were found to be binding stably with the target.

Levodopa, a pro drug of dopamine, is given to Parkinson’s patients because it can pass across the blood-brain barrier. Because Levodopa can convert to dopamine on either side of the blood-brain barrier, it is typically given along with a dopa decarboxylase inhibitor, such as carbidopa, to delay conversion until after it has passed the blood-brain barrier (Djamshidian and Poewe, 2016). Levodopa is converted to dopamine once it crosses the blood-brain barrier, supplementing the low endogenous levels of dopamine to treat Parkinson’s symptoms. The US-FDA authorized Sinemet, (Boshes, 1981; Djamshidian and Poewe, 2016), a Levodopa and carbidopa combination product, the first produced drug product to receive FDA approval. Droxidopa is a drug used to treat non-diabetic autonomic neuropathy, primary autonomic failure, and symptomatic neurogenic orthostatic hypotension (nOH) brought on by dopamine beta-hydroxylase deficiency. This drug is an orally active synthetic amino acid that is converted to norepinephrine by the enzyme aromatic L-amino acid decarboxylase (dopa-decarboxylase), was recently approved by the FDA for the short-term treatment of nOH. It is presumed to raise blood pressure by acting at the neurovascular junction to increase vascular tone (Kaufmann et al., 2015).

### Interactions of FDA-approved drugs with Rv3366

The eight drugs, namely, Kappadione, Pamidronic acid, Pyridoxal Phosphate, Cedrazuridine, Levodopa, Droxidopa, Pyrophosphoric acid and Zoledronic acid complexed with Rv3366 were simulated for 100ns. Initially, Sinefungin (SFG) the known pan inhibitor was superimposed on the catalytic site of Rv3366 using the Schrodinger suite using the structure of Rv3919c (PDB ID: 7CFE). Further it was docked at the same site and best docked pose interactions showed that SFG was making a hydrogen bond with Ser 8, Glu33, Leu63, Leu111, Ala112 and Asp113 are involved in other non-bonded interactions (Figures 1C, D). It had a docking score of −9.33 kcal/mol and MM/GBSA binding energy of −29.70 kcal/mol.

**FIGURE 2:**
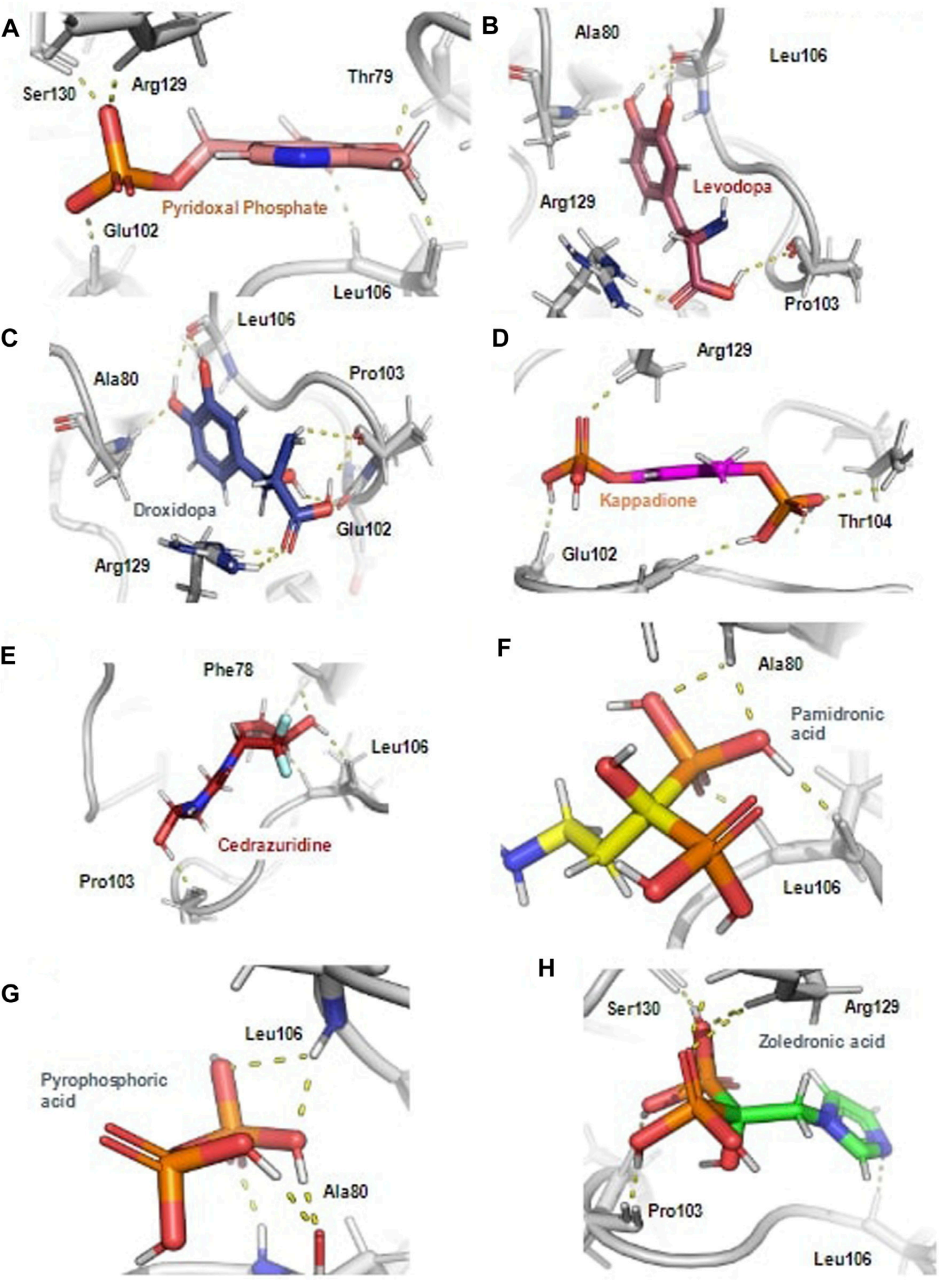
The hydrogen bond interaction of ligands with the Rv3366 in the docked pose. **(A)** The best binding pose and hydrogen bond interactions of Pyridoxal Phosphate with Rv3366 **(B)** Levadopa **(C)** Droxidopa **(D)** kappadione **(E)** Cedazuridine **(F)** Pamidronic acid **(G)** Pyro-phosphoric acid **(H)** Zoledronic acid.

Further, the top docked from the eight selected FDA drugs were also visualized, and it was observed that Pyridoxal Phosphate had the highest docking score of −9.41 kcal/mol, it’s MM/GBSA binding free energy was −54.4 kcal/mol and was making hydrogen bond with Phe78, Thr79, Glu102, Arg129 and Ser130 and was involved in other non-bonded interactions as shown in Figure 2A. Further, Levadopa had a docking score of −8.119 kcal/mol and MM/GBSA binding energy of −41.91 kcal/mol had hydrogen bonding with Thr79, Ala80, Pro103, Leu106 and Arg129 (Figure 2B). Likewise, Droxidopa had a docking score of −8.6 kcal and MM/GBSA −34.59 kcal/mol, and it was involved in the hydrogen bonding with Ala80, Glu102, Pro103, Leu106 and Arg129 (Figure 2C). Next, Kappadion had a docking score of −8.03 kcal/mol and MM/GBSA binding free energy of −56.16 kcal/mol, and it was making hydrogen bonding with Thr79, Pro103, Leu106 and Arg129 (Figure 2D). Cedazuridine had a docking score of −8.05 kcal and MM/GBSA binding free energy of −44.5 kcal/mol, making hydrogen bond interactions with Thr79, Ala80 and Pro103 (Figure 2E). Pamidronic acid had a docking score of −8.25 kcal/mol and MM/GBSA binding energy of −27.05 kcal/mol, and it was making hydrogen bond interactions with Ala80 and Leu106 (Figure 2F). Overall, it was found that all the molecules similar binding affinity as SFG and were interacting with common residues through hydrogen bonds and hydrophobic interactions as shown in Supplementary Figure S2 and could have a similar mode of inhibition.

### Levodopa and Droxidopa were found to have stable interactions with Rv3366

**FIGURE 3:**
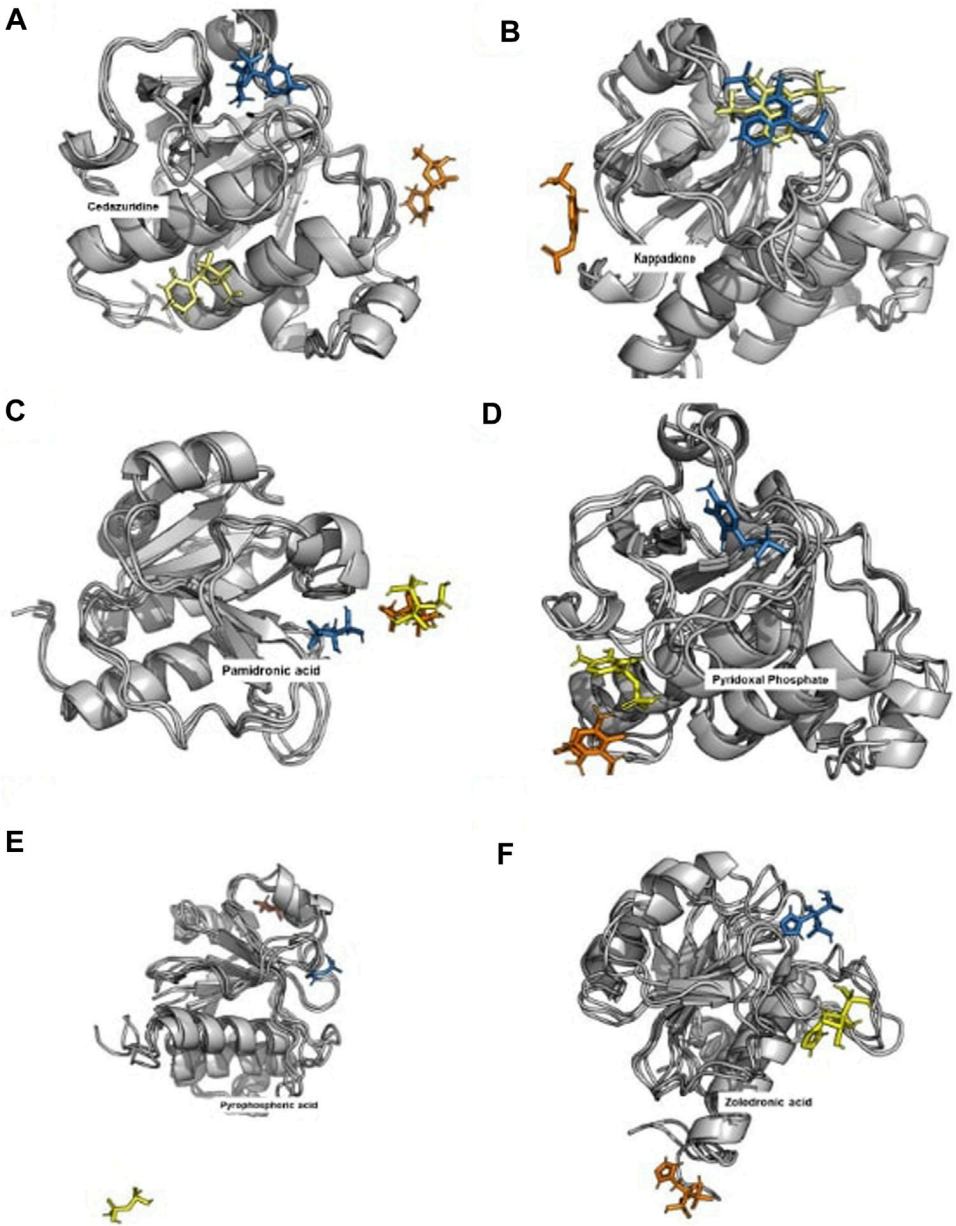
The binding pattern of unstable ligands during the 100 ns of simulations. The top docked compounds such as Cedazuridine, Kappadione, Pamidronic acid and Pyridoxal Phosphate could not bind stably at the binding pocket during the simulation. (The Blue color ligand shows the binding at 0th ns, Yellow color ligand shows the binding at 50th ns and Orange color ligand shows the binding at 100th ns. **(A)** Cedrazuridine **(B)** Kappadione **(C)** Pamidronic acid **(D)** Pyridoxal Phosphate **(E)** Pyrophosphoric acid **(F)** Zoledronic acid.

**FIGURE 4:**
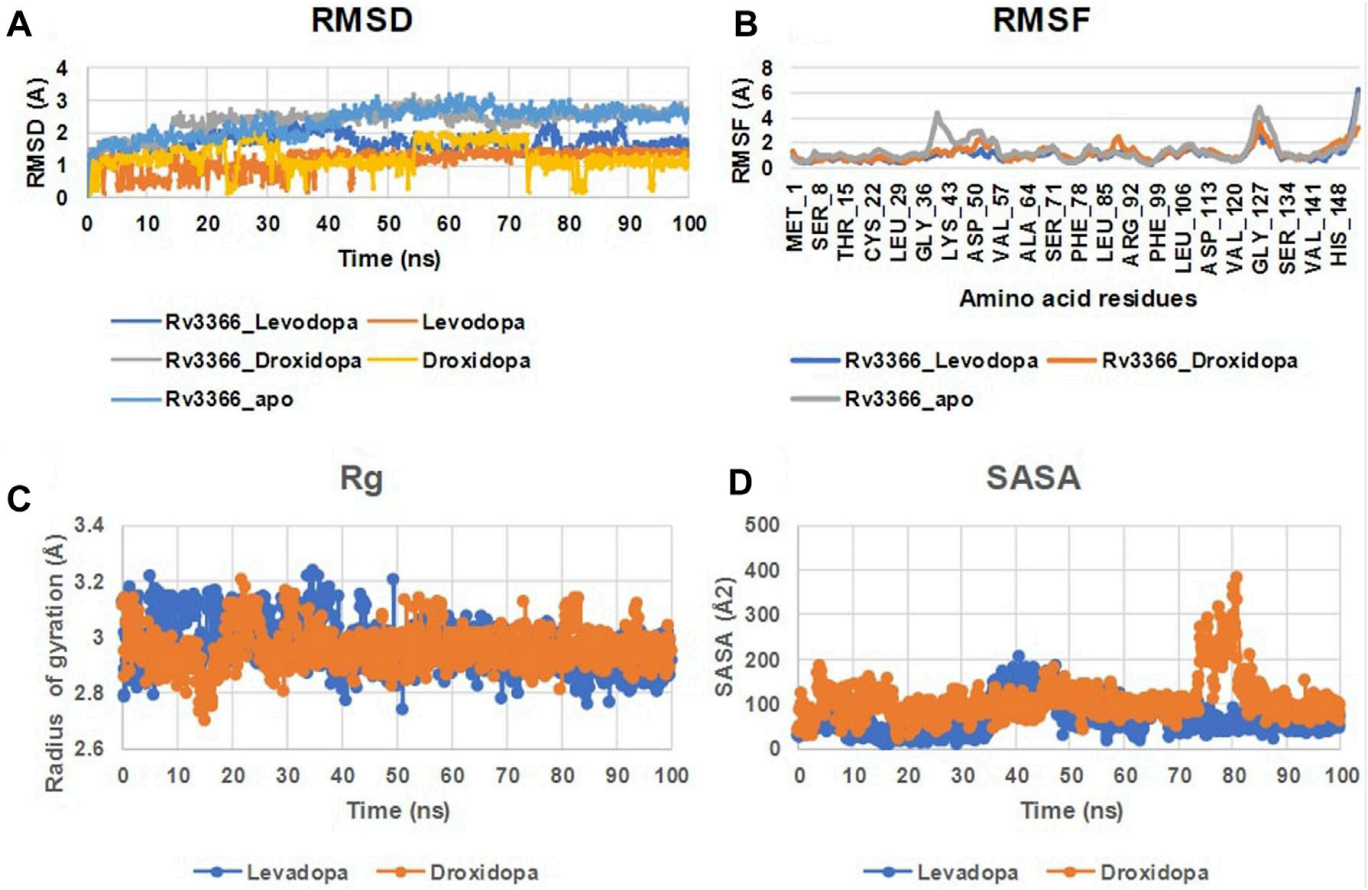
The MD simulation analysis of the protein-ligand complexes. **(A)** The RMSD plot of Rv3366 with ligands and ligands alone. **(B)** The RMSF calculations of the Rv3366-ligand complexes. **(C)** The radius of gyration of the Levodopa and Droxidopa through the simulation. **(D)** The solvent accesseble surface area for the ligands.

When the docked complexes were subjected to 100 ns explicit water classical MD simulation, it was found that Levodopa and Droxidopa could only interact stably with the protein target throughout the simulation time. The rest of the compound complexes were not able to bind stably within the binding pocket and they found to be translocating randomly around the protein in the trajectory (Figure 3). Therefore, two complexes (Levodopa and Droxidopa with Rv3366) were further taken to investigate their binding stability and crucial interactions at the binding site. The RMSD calculation showed that complexes, as well as the ligands alone, got stabilized within the first 30 ns of the simulation. The average deviation of Rv3366_apo was 2.36 ± 0.43 Å. While the average deviation of the Rv3366_Levodopa complex was 1.72 ± 0.28 Å, while Levodopa alone bound in the cavity had a deviation of 1.15 ± 0.33 Å, shows the binding of Levodopa made the complex more stable and compact. On the other hand, the average deviation of Rv3366_Droxidopa was 2.40 ± 0.37 Å, showing similar flexibility as the apo form of the protein. Droxidopa alone had an RMSD of 1.27 ± 0.41 Å in the binding cavity (Figure 4A).

Further, the RMSF of the complexes was calculated, and it did not show any abrupt fluctuation in the protein throughout the simulation time. It was observed that apo Rv3366 had a little more fluctuation (1.41 ± 0.91 Å) than the bound complexes. The average fluctuation in the Rv3366_Levodopa was 1.04 ± 0.66 Å and similar was for Rv3366_Droxidopa (1.19 ± 0.58 Å) (Figure 4B). Further, Levodopa and Droxidopa binding were also assessed through the radius of gyration (Harder, Damm et al.) and solvent-accessible surface area (SASA) calculation to observe their compactness and how well they fit inside the binding pocket, respectively. In Rg calculation, it was found that both the molecules had similar flexibility, Levadopa (2.97 ± 0.08 Å) and Droxidopa (2.96 ± 0.06 Å), and they are quite stable throughout the simulation (Figure 4C). In the case of SASA calculation, it was found that Levadopa (67 ± 35 Å2) was less accessible to solvent and more deeply bound at the binding site than Droxidopa (107.12 ± 45.15 Å2) (Figure 4D).

**FIGURE 5:**
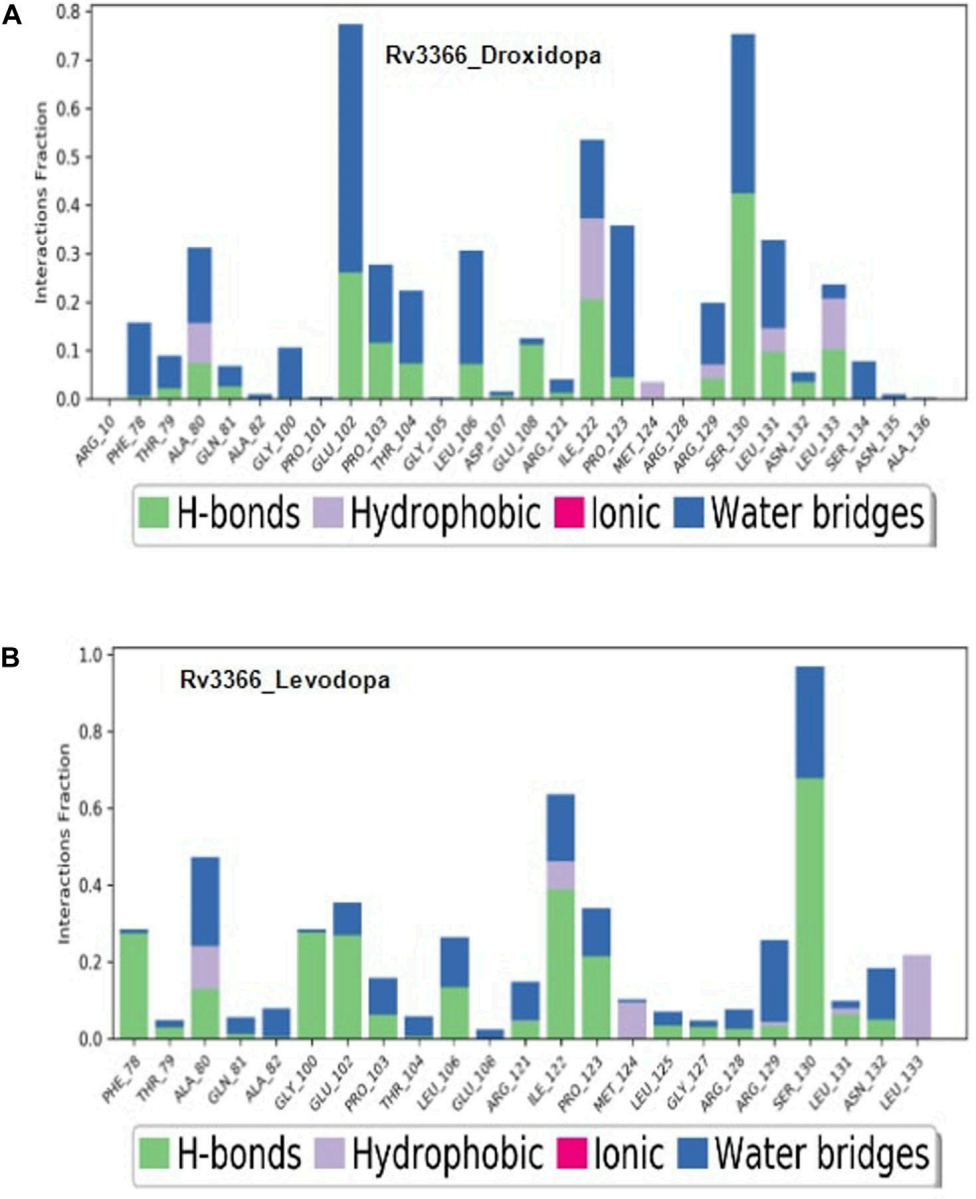
The simulation interaction diagram showing the kind of interactions and their occupancy throughout the simulation for Rv3366 **(A)** with Droxidopa **(B)** Levodopa.

Then, the simulation interactions were calculated to assess the crucial interactions, their types and occupancy of interactions. The results showed that both compounds had multiple interactions throughout the simulations, most of which involved hydrogen bonding. More specifically, Levodopa interacted critically (>40% of simulation time) with Ala80, Ile122 and ser130, while Droxidopa interacted significantly with Glu102, Ile122 and Ser130. Both of these ligands were interacting through Hydrogen bonding and water-mediated contacts. Overall, results suggested that both Levodopa and Droxidopa had stable binding at the Rv3366 catalytic pocket and residues Ala80, Ile122 and Ser130 are the critical residues at the binding site providing affinities to these drugs (Figures 5A, B).

## Discussion

The significance of RNA in protein production has been acknowledged for decades (Higgs and Lehman, 2015). RNA plays various crucial roles, such as mRNA for transcription, tRNA for translation, and rRNA for ribosomes. However, it also has additional functions, like controlling gene expression in eukaryotes (Zheng et al., 2015).

*M.tb*, a slow-growing pathogen, has limited RNA compared to DNA, with a significant portion being rRNA. RNA is essential for gene coding, regulation, and disease development (Burkert and Schumann, 2020). Alterations in bacterial rRNA, like methylation at position 518 of the 16S rRNA, can affect the behavior of *M.tb* and immune responses during infection (Demirci et al., 2010). Changes in rRNA modification can significantly impact ribosome assembly, function, and response to antibiotics. Certain rRNA methylations can either resist antibiotics or enhance drug binding and efficacy (Maguire, 2009). MTases, responsible for these methylations, are of interest due to their influence on pathogenicity and antibiotic resistance. Examples include erm (Rv 1988), which confers macrolide antibiotic resistance, and Rv2118c, a t-RNA MTase that affects bacterial virulence and proliferation. *M.tb* Rv2372c, an RsmE-like methyltransferase, methylates U1498 of the 16S rRNA, potentially aiding bacterial survival inside host macrophages. In case of recently reported DNA MTases, the immunomodulatory properties of the Rv1509 signature protein elicit an immunological memory response, which may have consequences for serodiagnosis and the development of TB vaccines (Manjunath et al., 2021). Another MTase, Rv2966c, may methylate N2-G966 of the 16S rRNA and benefit bacteria under stress, like *M.tb* inside human macrophages. In *M.tb*, MTases represent 3% of the genome despite the reductive evolution of *M.tb* (Ahmed et al., 2008). Overall, MTases are promising targets for developing new drugs to disrupt *M.tb*’s core mechanisms and pathogenesis (Varshney et al., 2004; Madsen et al., 2005; Kumar et al., 2011; Kumar et al., 2014; Grover et al., 2016).

SpoU is a probable RNA MTase and is vastly studied in other prokaryotes where it is shown to project changes in RNA. For example, in *Escherichia coli spoU* that is similar to 2′-O- methyltransferase modifies tRNA (Gm18) that is completely absent in the *spoU* mutant. Also, in yeast (*Saccharomyces cerevisiae)* among three RNA 29-O-ribose methylases, *spoU* methylates tRNA (G18) which was not detected in *spoU* disrupted strains. In *Thermus thermophilus*, a heat-favourable microbe, certain t-RNAs often have a chemical modification called 2′-O-methylguanosine at position 18 in the D-loop, and this alteration is added to the RNA molecule after it has been transcribed by an enzyme known as tRNA (Gm18) methyltransferase. The genome of *Aquifex aeolicus*, a hyper-thermophile eubacterium, encodes a novel type II Gm-methylase gene or t = RNA (guanosine-2′)-methyltransferase (Gm-methylase) that catalyzes the transfer of a methyl group from S-adenosyl-l-methionine to 2′-OH of G18 in the D-loop of tRNA. Evidently, this Gm-methylase factor has the potential to alter the structure of tRNAs. (Gustafsson et al., 1996; Persson et al., 1997; Cavaillé et al., 1999; Hori et al., 2002; Hori et al., 2003). Also it has been reported that SpoU associates with rifampicin and ethambutol which is a case of artefactual cross-resistance (Consortium, 2022). It is important to consider the potential for presuming a drug target that could obstruct connections with these enzymes in order to perhaps disrupt host-pathogen interactions. For example, the pathogens developed in phagosomes and phagolysosomes manipulate or use the common host as well as pathogen targets to take advantage of the host immune system. These shared molecules or pathways may serve as broad-spectrum therapeutic targets in the development of drugs to combat infectious illnesses brought on by various intracellular infections (Selvapandiyan et al., 2023). This might lessen the severity of an illness or facilitate the immune system’s ability to fight it. Inhibiting acetylated region of the bacteria may be used as a weapon to prevent the growth of bacteria that are resistant to antibiotics because increasing data suggest that it plays a role in the development of antibiotic resistance. *In silico* approach was used to observe SpoU binding with FDA approved compound so that a novel drug target can be proposed to fight against drug resistant *M.tb*. The compounds were initially prepared and filtered in the workflow based on the Lipinski rule of five. Further, based on the virtual screening, the top 50 FDA-approved drugs were screened as top docked molecules against both targets. It was observed that Levodopa and Droxidopa could only interact stably with the protein target among other drugs. Levodopa is a pro-drug of dopamine that is given to Parkinson’s patients because it can pass across the blood-brain barrier. Whereas Droxidopa is a drug used to treat non-diabetic autonomic neuropathy, primary autonomic failure, and symptomatic neurogenic orthostatic hypotension (nOH) brought on by dopamine beta-hydroxylase deficiency. The binding energy of Levodopa with SpoU protein was −41.91 kcal/mol and with Droxidopa it was −34.59 kcal/mol. Hydrogen-bonds play a critical role in determining the specificity of ligand binding. Levodopa exhibited four hydrogen bonds in the best-docked pose with Ala80, Pro103, Leu106 and Arg129 and Droxidopa made five hydrogen bonds in the best binding pose with Ala80, Glu102, Pro103, Leu106 and Arg129. Both of the compounds showed efficient docking score which projects towards the potential of a novel drug target against TB.

## Conclusion

This study highlights the role of *M.tb* RNA MTases as important players in *M.tb* cell growth and also suggests that Rv3366 could serve as a valuable drug target for anti-TB drug development. The two FDA approved drugs; Levodopa and Droxidopa which are involved in the treatment of neurological and autonomic disorders respectively, were observed to strongly bind within the active site of Rv3366. The formation of stable hydrogen and hydrophobic interactions between Levodopa-Rv3366 and Droxidopa-Rv3366 complexes reflects their nature as probable inhibitors of this crucial enzyme. However, the true inhibitory effect of these two drugs needs to be critically evaluated via *in vitro* studies. The results described here represent the groundwork done towards the identification of new repurposed drugs that could further be investigated in preclinical and clinical settings. We strongly believe that the available pharmacokinetics and pharmacodynamics data on Levodopa and Droxidopa would cut short the time required for drug safety validations in humans and may speed up the anti-TB drug repurposing process. Moreover, this study sheds light on how using computer-based techniques is crucial in pinpointing potential drug targets and finding new uses for existing drugs to combat tuberculosis. By combining the strengths of scientific knowledge and inventive approaches, we aim to lessen the global burden of TB and ultimately safeguard lives across the world.

## Data Availability

The original contributions presented in the study are included in the article/Supplementary Material, further inquiries can be directed to the corresponding authors.
