# Supplementary material for: Computational analysis of RNA methyltransferase Rv3366 as a potential drug target for combating drug-resistant Mycobacterium tuberculosis
Source: Front Mol Biosci. 2024 Jan 11;10:1348337. doi: 10.3389/fmolb.2023.1348337 (PMC10808684; doi:10.3389/fmolb.2023.1348337)
Supplement: Supplementary file 1 [file Table1.DOCX]

Supplementary table (Rv3366):

| Generic name | Traditional IUPAC | Docking score (kcal/mol) | MM/GBSA (kcal/mol) | Function |
| --- | --- | --- | --- | --- |
| Kappadione | tetrasodium 3-methyl-4-(phosphonatooxy)naphthalen-1-yl phosphate | -8.032 | -56.16 | Kappadione is a Vitamin K derivative (chemically, it is menadiol sodium diphosphate), previously approved by FDA prior to 1982 and marketed by Lilly Marketing for this drug has been discontinued and is not available in North America 3. It has been found to have carcinogenic potential in mammalian cells as well as cytotoxic properties 4. Studies involving the active metabolite of this formulation, menadione, showed oocyte toxicity in a study of mice 4. |
| Menadiol diphosphate | [3-methyl-4-(phosphonooxy)naphthalen-1-yl]oxyphosphonic acid | -8.135 | -55.82 | This drug entry is a stub and has not been fully annotated. It is scheduled to be annotated soon. Menadiol sodium diphosphate is a water-soluble vitamin K analogue. The presence of vitamin K is essential for the formation within the body of prothrombin, factor VII, factor IX and factor X. Lack of vitamin K leads to increased tendency to haemorrhage. |
| Pyridoxal phosphate | pyridoxal phosphate | -9.413 | -54.4 | This is the active form of vitamin B6 serving as a coenzyme for synthesis of amino acids, neurotransmitters (serotonin, norepinephrine), sphingolipids, aminolevulinic acid. During transamination of amino acids, pyridoxal phosphate is transiently converted into pyridoxamine phosphate (pyridoxamine). |
| Imidurea | imidazolidinyl urea | -7.46 | -50.89 | Imidurea is an antimicrobial preservative used in cosmetics. It acts as a formaldehyde releaser. |
| Epinephrine | epinephrine | -7.515 | -45.35 | Epinephrine, also known as adrenaline, is a hormone and neurotransmitter and produced by the adrenal glands that can also be used as a drug due to its various important functions. In general, the most common uses of parenteral epinephrine are to relieve respiratory distress due to bronchospasm, to provide rapid relief of hypersensitivity (anaphylactic or anaphylactoid) reactions to drugs, animal serums and other allergens, and to prolong the action of infiltration anesthetics 20. In addition to the above functions, epinephrine is the primary drug administered during cardiopulmonary resuscitation (CPR) to reverse cardiac arrest 3, 4. It can be used in severe cases of croup 14. |
| Norepinephrine | norepinephrine | -8.484 | -44.98 | Precursor of epinephrine that is secreted by the adrenal medulla and is a widespread central and autonomic neurotransmitter. Norepinephrine is the principal transmitter of most postganglionic sympathetic fibers and of the diffuse projection system in the brain arising from the locus ceruleus. It is also found in plants and is used pharmacologically as a sympathomimetic. |
| Cedazuridine | (4R)-1-[(2R,4R,5R)-3,3-difluoro-4-hydroxy-5-(hydroxymethyl)oxolan-2-yl]-4-hydroxy-1,3-diazinan-2-one | -8.035 | -44.5 | Myelodysplastic syndromes (MDS) are a group of hematopoietic neoplasms that give rise to variable cytopenias progressing to secondary acute myeloid leukemia (sAML), which is invariably fatal if untreated.1,2,3 Hypomethylating agents such as decitabine and azacitidine are used to treat MDS through inducing DNA hypomethylation and apoptosis of cancerous cells.7,8 Although effective, these compounds are rapidly metabolized by cytidine deaminase (CDA) prior to reaching systemic circulation when administered orally, necessitating intramuscular or intravenous administration routes. |
| Arbutin | Î²-arbutin | -7.809 | -44.29 | Extracted from the dried leaves of bearberry plant in the genus Arctostaphylos and other plants commonly in the Ericaceae family, arbutin is a beta-D-glucopyranoside of Hydroquinone. It is found in foods, over-the-counter drugs, and herbal dietary supplements. It has also been used as an anti-infective for the urinary system as well as a diuretic 6. Arbutin is available in both natural and synthetic forms; it can be synthesized from acetobromglucose and Hydroquinone 6. Arbutin is a competitive inhibitor of tyrosinase (E.C.1.14.18.1) in melanocytes 1, and the inhibition of melanin synthesis at non-toxic concentrations was observed in vitro. Arbutin was shown to be less cytotoxic to melanocytes in culture compared to Hydroquinone 5. |
| Pyridoxine | pyridoxine | -7.617 | -43.51 | Pyridoxine is the 4-methanol form of vitamin B6, an important water-soluble vitamin that is naturally present in many foods. As its classification as a vitamin implies, Vitamin B6 (and pyridoxine) are essential nutrients required for normal functioning of many biological systems within the body. Pyridoxine is used medically for the treatment of vitamin B6 deficiency and for the prophylaxis of isoniazid-induced peripheral neuropathy (due to Isoniazid's mechanism of action which competitively inhibits the action of pyridoxine in the above-mentioned metabolic functions). It is also used in combination with Doxylamine (as the commercially available product Diclectin) for the treatment of nausea and vomiting in pregnancy. |
| Levodopa | levodopa | -8.119 | -41.91 | Levodopa is a prodrug of dopamine that is administered to patients with Parkinson's due to its ability to cross the blood-brain barrierLabel. Levodopa can be metabolised to dopamine on either side of the blood-brain barrier and so it is generally administered with a dopa decarboxylase inhibitor like carbidopa to prevent metabolism until after it has crossed the blood-brain barrier. Once past the blood-brain barrier, levodopa is metabolized to dopamine and supplements the low endogenous levels of dopamine to treat symptoms of Parkinson'sL |
| Telbivudine | telbivudine | -7.499 | -41.89 | Telbivudine is a synthetic thymidine nucleoside analog with specific activity against the hepatitis B virus. Telbivudine is orally administered, with good tolerance, lack of toxicity and no dose-limiting side effects. |
| Pirbuterol | pirbuterol | -7.64 | -41.38 | Pirbuterol is a beta-2 adrenergic bronchodilator. In vitro studies and in vivo pharmacologic studies have demonstrated that pirbuterol has a preferential effect on beta-2 Adrenergic receptors compared with isoproterenol. The pharmacologic effects of beta adrenergic agonist drugs, including pirbuterol, are at least in proof attributable to stimulation through beta adrenergic receptors of intracellular adenyl cyclase, the enzyme which catalyzes the conversion of adenosine triphosphate (AlP) to cyclic-3† ,5†-adenosine monophosphate (c-AMP). Increased c-AMP levels are associated with relaxation of bronchial smooth muscle and inhibition of release of mediators of immediate hypersensitivity from cells, especially from mast cells. |
| Fluorodopa (18F) | 6-(18F)Fluoro-L-DOPA | -7.388 | -39.46 | Fluorodopa F 18 is a fluorinated analog of levodopa used as a diagnostic agent for positron emission tomography (PET) in the evaluation of Parkinsonian syndromes.1 Fluorodopa F 18 PET is used adjunctly with other diagnostic investigations and serves primarily to visualize dopaminergic nerve terminals in the striatum. |
| Tyrosine | ethane | -7.486 | -36.49 | Tyrosine is a non-essential amino acid. In animals it is synthesized from phenylalanine. It is also the precursor of epinephrine, thyroid hormones, and melanin. |
| alpha-Arbutin | Î±-arbutin | -7.5 | -35.04 | This drug entry is a stub and has not been fully annotated. It is scheduled to be annotated soon. Extracted from plants such as bearberries, blueberries, and cranberries, alpha arbutin is a safe skin brightening ingredient which helps to fade scars and pigmentation left behind by breakouts and sun damage. |
| Droxidopa | (2S,3R)-2-amino-3-(3,4-dihydroxyphenyl)-3-hydroxypropanoic acid | -8.653 | -34.59 | Droxidopa is a precursor of noradrenaline that is used in the treatment of Parkinsonism. It is approved for use in Japan and is currently in trials in the U.S. The racaemic form (dl-threo-3,4-dihydroxyphenylserine) has also been used, and has been investigated in the treatment of orthostatic hypotension. There is a deficit of noradrenaline as well as of dopamine in Parkinson's disease and it has been proposed that this underlies the sudden transient freezing seen usually in advanced disease. |
| Oxidronic acid | HMDP | -8.6 | -28.49 | This drug entry is a stub and has not been fully annotated. It is scheduled to be annotated soon. Oxidronic acid is a diagnostic skeletal imaging agent used to demonstrate areas of altered osteogenesis in adult and pediatric patients. |
| Pamidronic acid | pamidronate | -8.259 | -27.05 | Pamidronic acid is a second generation, nitrogen containing bisphosphonate similar to neridronic acid and alendronic acid.2 Pamidronic acid was first described in the literature in 1977.9 The second generation bisphosphonates are less common as third generation bisphosphonates, such as ibandronic acid, zoledronic acid, minodronic acid, and risedronic acid are becoming more popular. |
| Technetium Tc-99m oxidronate | HMDP (â¹â¹Tc)technetium | -8.564 | -26.24 | Technetium Tc-99m oxidronate, also known as 99mTc-methylene diphosphonate, is a radiopharmaceutical agent. A radiopharmaceutical is defined as a medicinal formulation containing radioisotopes that are used in major clinical areas for diagnosis and/or therapy.2 The radiopharmaceuticals based on technetium-99m are widely used for diagnostic purposes because 99mTc has a versatile chemistry which allows it to produce an extense variety of complexes with specific characteristics.3 These complexes are formed by the binding of 99mTc to metal atoms of an organic molecule. The group oxidronate falls into the category of diphosphonates whose structure allows them to bind to calcium. |
| Pyrophosphoric acid | pyrophosphoric acid | -8.816 | -26.19 | This drug entry is a stub and has not been fully annotated. It is scheduled to be annotated soon. Pyrophosphoric acid is an ingredient of a radiopharmaceutical used to visualize bone abnormalities and cardiovascular abnormalities and also used as an ingredient in some products to prevent iron deficiency anemia. |
| Technetium Tc-99m pyrophosphate | (99Tc)technetium(4+) ion phosphonooxyphosphonate | -8.816 | -26.08 | A radionuclide imaging agent used primarily in scintigraphy or tomography of the heart to evaluate the extent of the necrotic myocardial process. It has also been used in noninvasive tests for the distribution of organ involvement in different types of amyloidosis and for the evaluation of muscle necrosis in the extremities. |
| Zoledronic acid | zoledronate | -7.844 | -22.33 | Zoledronic acid, or CGP 42'446,8 is a third generation, nitrogen containing bisphosphonate similar to ibandronic acid, minodronic acid, and risedronic acid.5 Zoledronic acid is used to treat and prevent multiple forms of osteoporosis, hypercalcemia of malignancy, multiple myeloma, bone metastases from solid tumors, and Paget’s disease of bone. |
