# Supplementary material for: Computational analysis of RNA methyltransferase Rv3366 as a potential drug target for combating drug-resistant Mycobacterium tuberculosis
Source: Front Mol Biosci. 2024 Jan 11;10:1348337. doi: 10.3389/fmolb.2023.1348337 (PMC10808684; doi:10.3389/fmolb.2023.1348337)
Supplement: Supplementary file 2 [file DataSheet1.docx]

**Supplemetary Figures**


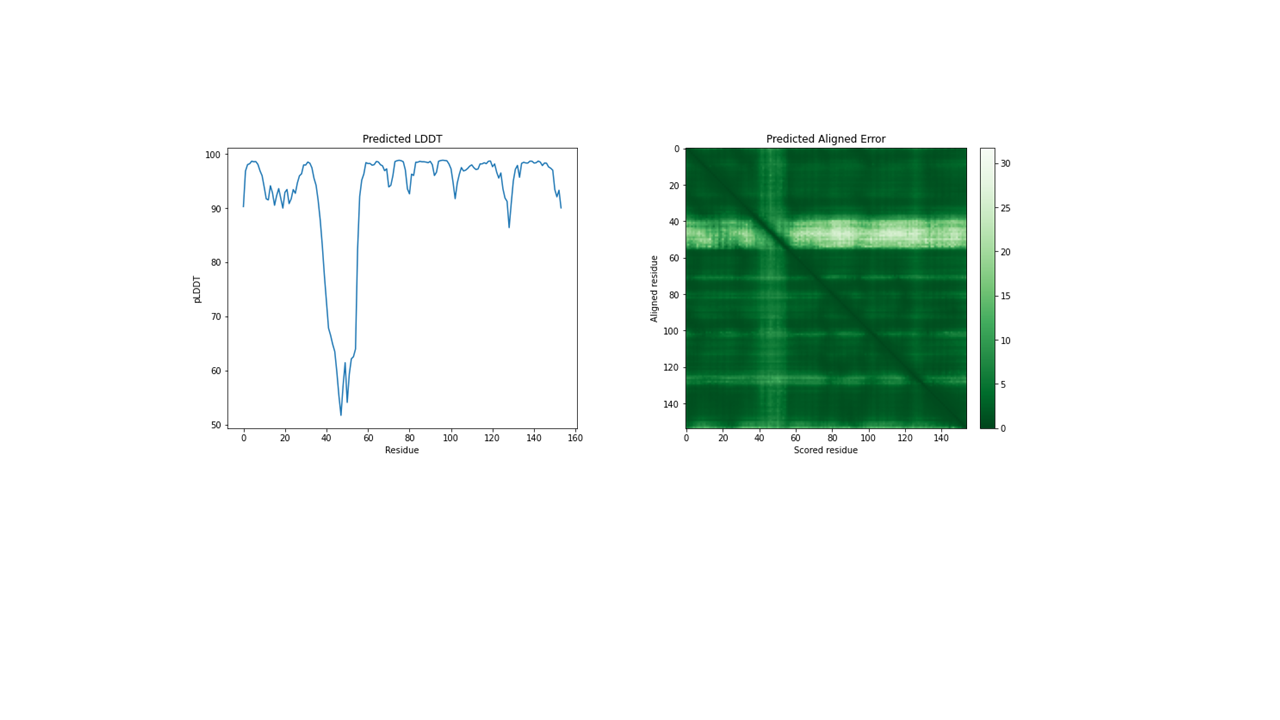


**Supplementary Figure 1. The pLDDT score and Predicted Aligned error matrix of the modelled protein (Rv3366).**

**
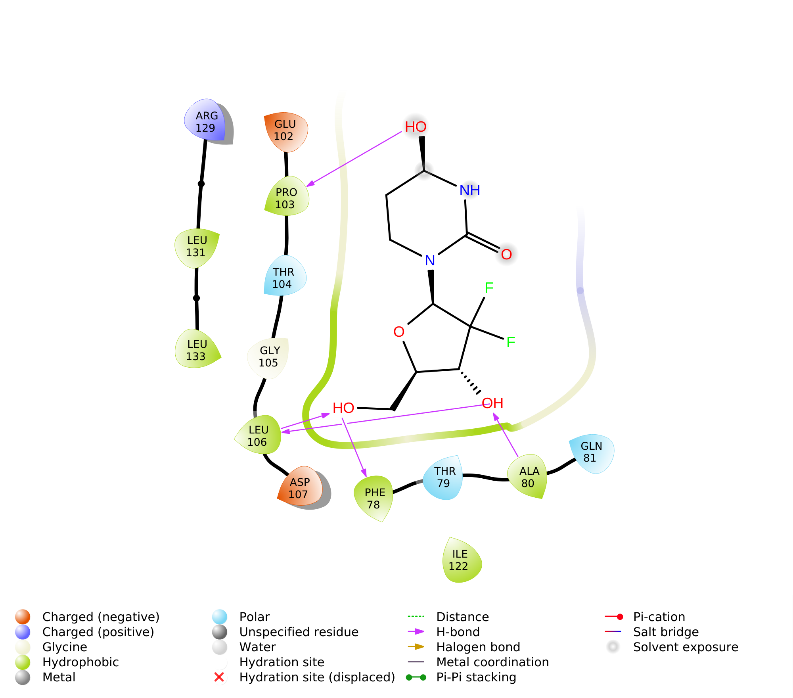

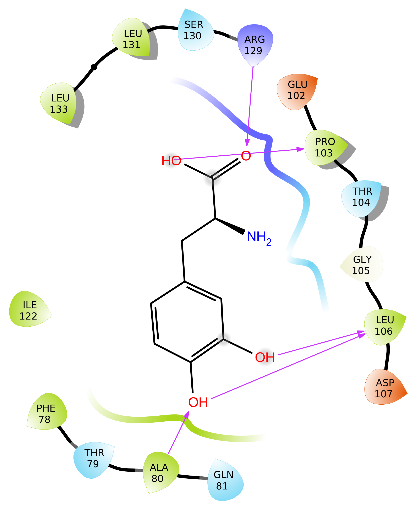

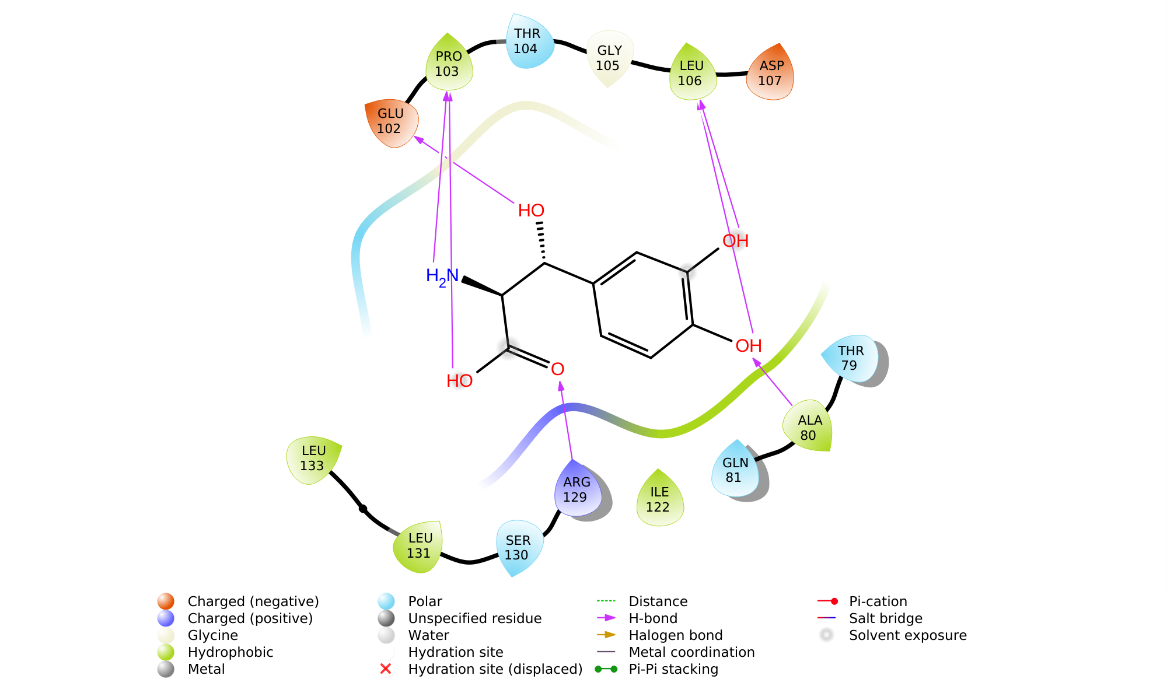

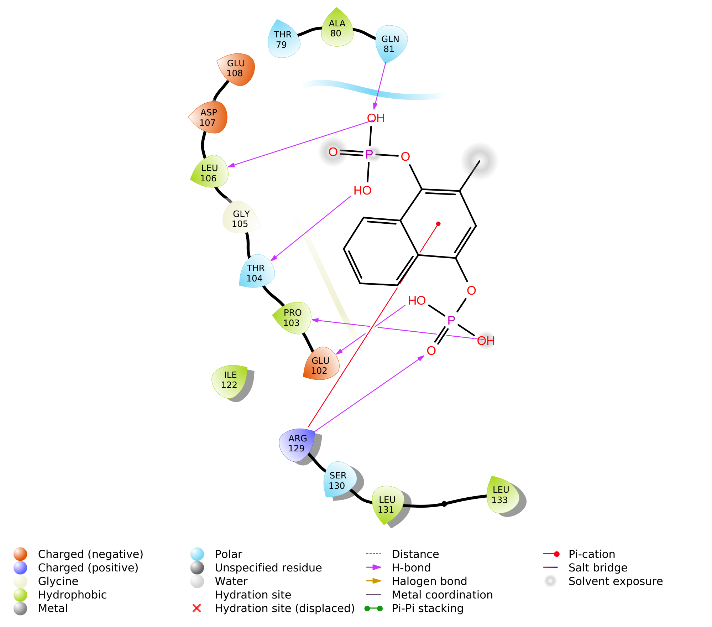
**

D

C

B

A

**
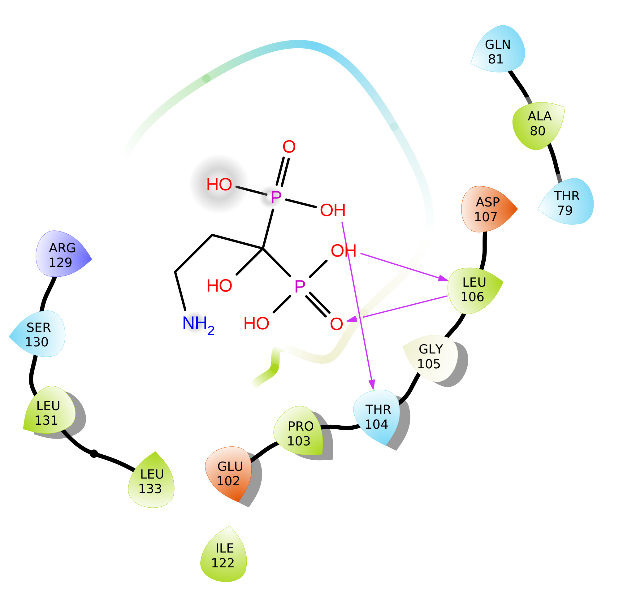

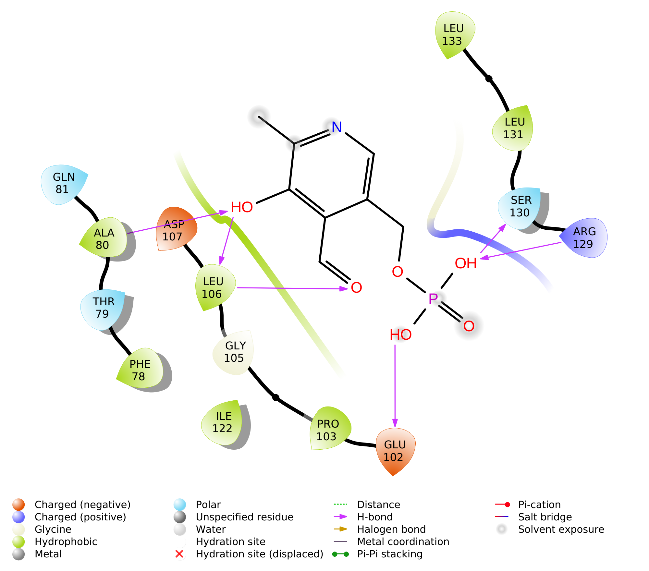
**

F

E

**
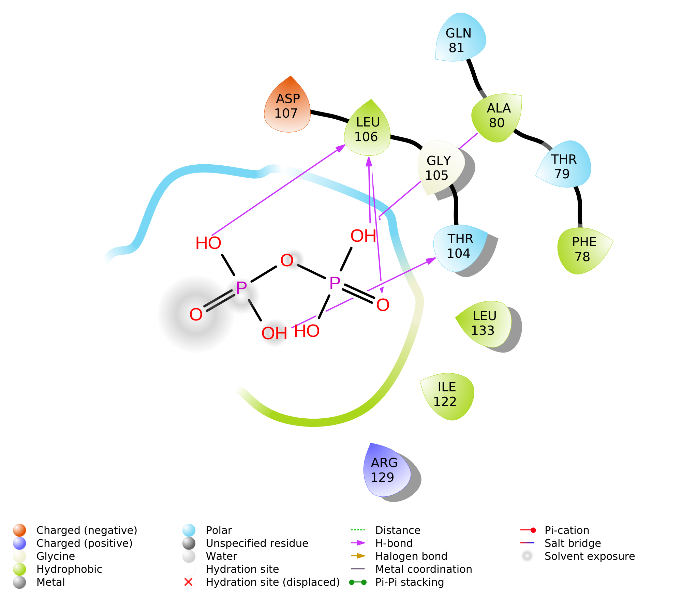

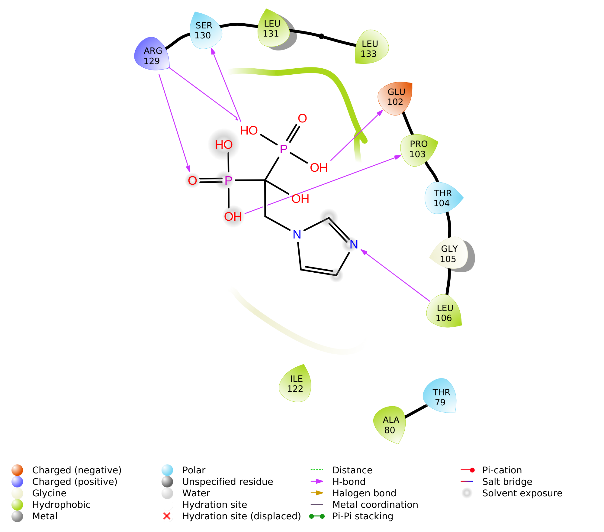
**

H

G

**Supplementary Figure 2. The 2D interaction diagram of the best docked pose ligands with Rv3366. (A) Cedrazuridine (B) levodopa (C) Droxidopa (D) Kappadione (E) Pamidronic acid (F) Pyridoxal Phosphate (G) Pyrophosphoric acid (H) Zoledronic acid.**
